# Case Reports: Emery-Dreifuss Muscular Dystrophy Presenting as a Heart Rhythm Disorders in Children

**DOI:** 10.3389/fcvm.2021.668231

**Published:** 2021-05-07

**Authors:** Tatiana Kovalchuk, Elena Yakovleva, Svetlana Fetisova, Tatiana Vershinina, Viktoriya Lebedeva, Tamara Lyubimtseva, Dmitriy Lebedev, Lubov Mitrofanova, Anton Ryzhkov, Polina Sokolnikova, Yuliya Fomicheva, Alexandra Kozyreva, Sergey Zhuk, Natalia Smolina, Anna Zlotina, Tatiana Pervunina, Anna Kostareva, Elena Vasichkina

**Affiliations:** ^1^World-Class Research Centre for Personalized Medicine, Almazov National Medical Research Centre, Saint Petersburg, Russia; ^2^Institute of Heart and Vessels, Almazov National Medical Research Centre, Saint Petersburg, Russia; ^3^Pathology Unit, Almazov National Medical Research Centre, Saint Petersburg, Russia; ^4^Radiology Unit, Almazov National Medical Research Centre, Saint Petersburg, Russia; ^5^Institute of Molecular biology and Genetics, Almazov National Medical Research Centre, Saint Petersburg, Russia; ^6^Institute of Perinatology and Pediatrics, Almazov National Medical Research Centre, Saint Petersburg, Russia; ^7^Department of Women's and Children's Health and Center for Molecular Medicine, Karolinska Institutet, Solna, Sweden

**Keywords:** Emery-Dreifuss muscular dystrophy, children, atrial tachycardia, atrial fibrillation, cardiomyopathy, *LMNA*, *EMD* (emerin), pacemaker implantation

## Abstract

Emery-Dreifuss muscular dystrophy (EDMD) is inherited muscle dystrophy often accompanied by cardiac abnormalities in the form of supraventricular arrhythmias, conduction defects and sinus node dysfunction. Cardiac phenotype typically arises years after skeletal muscle presentation, though, could be severe and life-threatening. The defined clinical manifestation with joint contractures, progressive muscle weakness and atrophy, as well as cardiac symptoms are observed by the third decade of life. Still, clinical course and sequence of muscle and cardiac signs may be variable and depends on the genotype. Cardiac abnormalities in patients with EDMD in pediatric age are not commonly seen. Here we describe five patients with different forms of EDMD (X-linked and autosomal-dominant) caused by the mutations in *EMD* and *LMNA* genes, presented with early onset of cardiac abnormalities and no prominent skeletal muscle phenotype. The predominant forms of cardiac pathology were atrial arrhythmias and conduction disturbances that progress over time. The presented cases discussed in the light of therapeutic strategy, including radiofrequency ablation and antiarrhythmic devices implantation, and the importance of thorough neurological and genetic screening in pediatric patients presenting with complex heart rhythm disorders.

## Introduction

Emery-Dreifuss muscular dystrophy (EDMD) is a group of inherited muscle-joint-cardio syndromes (1). The cardiac involvement in EDMD can be severe and life-threatening (2). Various genetic backgrounds contribute in different ways to the broad spectrum of cardiac manifestations, mainly in the form of conduction disorders, supraventricular arrhythmias and more rarely functional and structural abnormalities of the heart (3).

EDMD is a rare disease. The general prevalence of EDMD is 0.39–1 per 100,000, with significant heterogeneity of estimates; in the pediatric population −0.22 per 100,000 (4–6). The classic clinical triad includes early joint contractures (elbows, neck, ankles, and spine), slowly progressive muscle weakness/atrophy and cardiac abnormalities, but the clinical course and sequence of symptoms depend on the genotype. The severity of cardiac involvement does not correspond to the progression of muscular weakness (3). In the case of EDMD1, associated with mutations in the *EMD* gene, the contractures frequently emerge in the first decade of life, become more significant during the growth spurt followed by muscle atrophy and weakness in the second decade of life and usually precede cardiac phenotype. In EDMD2, caused by mutations in the *LMNA* gene, symptoms varied widely from a mild phenotype with a later onset and slow progression to a severe one with life-threatening complications. Lack of information for the reported cases EDMD3, EDMD4, and EDMD5 linked to the genes encoding proteins of nuclear envelope does not allow to define a unified clinical picture of each of those subtypes (7, 8).

Cardiac involvement in EDMD usually follows muscle phenotypes and predominantly presents after the second decade of life (9, 10). After the diagnosis is established due to typical muscular manifestation, the cardiac abnormalities are screened in the frame of expected disease phenotype (11). Several cases of early cardiac debut preceding muscle dystrophy have been reported, and even isolated cardiac involvement has been described; still, these cases remain scarce and atypical (10, 12–15). The cardiac disease commonly manifests from atrial arrhythmias and conduction disturbances. Syncope and sudden cardiac death (SCD), caused by complete heart block or ventricular tachyarrhythmia, can also occur, and single cases of left ventricular non-compaction have been described (16, 17). Systolic dysfunction and dilated cardiomyopathy found in a minority of patients and are mainly associated with autosomal-dominant disease (AD-EDMD) due to *LMNA*-mutations (18–22). Creatine kinase (CK) level can range from normal to 15 times the upper limit, without direct correlation with muscular and cardiac involvement, so in patients with severe cardiac phenotype CK levels could remain normal (23).

The genetic spectrum of EDMD includes mutations in *EMD, LMNA, SYNE1, SYNE2, FHL1, TMEM43, SUN1, SUN2*, and *TTN* genes (5, 24–28). These genes mainly encode for the nuclear envelope proteins, which give rise to the term “nuclear envelopathies” (5, 23, 29). An exception is FHL1 protein which localizes to the sarcomere and the sarcolemma but may also shuttle between cytosolic and nucleoplasmic fraction, thus becoming a part of the nuclear envelope (30). Mutations in *LMNA* and *EMD* are the most common causes of EDMD, and together they account for about 36% of the cases (5). Thus, for EDMD, there are still a number of undetected causative genes (30). An AD-EDMD mainly arises from mutations in *LMNA*, which contribute to ~28% of the cases. In *LMNA*-associated EDMD, males and females are equally affected and left ventricular dysfunction is more commonly observed as well as ventricular arrhythmias and SCD (9, 19, 20). Several cases of *LMNA*-associated autosomal-recessive form of EDMD presented in pediatric patients have also been described (31, 32). The X-linked form is usually caused by mutations in the *EMD* gene (8%) and, rarely, in the *FHL1* gene (2%) and predominantly affects males with rare cases of disease manifestation in female carriers (18). Mutations in the *EMD* gene occur sporadically and rarely, but mutations in the *LMNA* gene are increasingly identified (23).

Due to the typical presentation during the second-third decades of life and a debut from muscle symptoms, there are only several reports on EDMD presented in children with the isolated cardiac phenotype (13, 14, 33–35). Here we describe five cases of EDMD1 and EDMD2 with a cardiac manifestation in childhood and discuss the need for target screening of neuromuscular phenotypes in children with unexplained atrial dysfunction, conduction abnormalities, and ventricular arrhythmias.

## Materials and Methods

Patients were examined between 2009 and 2020 in tertiary pediatric cardiac care center – Almazov National Medical Research Centre, St. Petersburg, Russia. All data, including the clinical history, case notes, reports of instrumental methods and surgical protocols were extracted from paper and electronic databases. Clinical examination included physical, 12-lead electrocardiography (ECG), Holter monitoring (HM), transthoracic echocardiography, neurological examination and laboratory tests. Additionally, we performed CMR, electrophysiological study (EPS) and electroneuromyography (ENMG). One patient underwent an endomyocardial biopsy to exclude inflammatory heart disease.

## Genetic Analysis

Target sequencing was performed using a panel of 108 or 172 genes, as previously described (36). For Patients 1 and 2, a targeted panel of 108 cardiomyopathy-associated genes has been initially studied using Haloplex Target Enrichment System (Agilent, Waldbronn, Germany) with an Illumina MiSeq instrument (for gene list, see Supplementary Table 1). For Patients 3–5, a targeted panel of 172 cardiomyopathy-associated genes was studied using the SureSelect Target Enrichment System (Agilent; Waldbronn, Germany) (see Supplementary Table 2). Data processing and variant calling were performed according to GATK BestPractice recommendations (Broad Institute, Cambridge, MA, USA) using hg19 and hg38 human genome references. For variant validation, bidirectional Sanger sequencing was performed using ABI 3500 machine (Applied Biosystems). All novel and previously reported variants of interest with a frequency below 0.01% were classified according to the recommendations of American College of Medical Genetics (37). The described variants were submitted to Gene bank repository under submission numbers SCV001548550 - SCV001548554.

## Ethical Considerations

The study was performed according to the Declaration of Helsinki, and approval was obtained from the local ethical committee of Almazov National Medical Research Centre. Written informed consents were obtained from the parents of the minor prior to investigation.

## Results

We observed five patients with EDMD and cardiac symptoms in childhood: three with EDMD1 and two with EDMD2. All patients were males. The mean age of cardiac manifestation was 13.2 ± 3.11 (from 9 to 16 y.o.). The mean follow-up period was 7.4 ± 2.6 years. All patients had sinus node dysfunction and four out of five - atrioventricular block (AVB). The leading arrhythmic phenotypes included various types of supraventricular arrhythmias: multifocal atrial tachycardia (mAT) (*n* = 4), premature atrial captures (PACs) (*n* = 4), atrial flutter (AF) (*n* = 3), atrial fibrillation (AFib) (*n* = 3) and AV nodal recurrent tachycardia (AVRNT). Arrythmias were the first manifestation in four patients. Patients predominantly complained of palpitation (*n* = 4), dizziness (*n* = 1), fatigue, and reduced physical tolerance (*n* = 2) (Table 1).

**Table 1:** Clinical and genetic characteristics of patients affected by EDMD and presented with heart rhythm disorders.

	**Patient 1**	**Patient 2**	**Patient 3**	**Patient 4**	**Patient 5**
Mutation variant	**EMD** NM_000117.3:c.631delC (pArg211ValfsTer26)	**EMD** NM_000177.3:c.449+1G>A	**EMD** NM_000117.3:c.173C>T (p.Ser58Phe) rs781797234	**LMNA** NM_170707.4:c.746G>A (p.Arg249Gln) rs59332535	**LMNA** NM_170707.3:c.305T>C (p.Leu102Pro) rs1553262
Age at first cardiac manifestation	14 y.o.	11 y.o. (possibly earlier)	16 y.o.	16 y.o.	9 y.o.
Heart rhythm disorders	AT, AF, AFib, PACs, AVNRT, SSNS, AVB II	AT, PACs, SSNS	SB, AVB I, II	AFib, AF, PACs, PVCs, SAB II, AVB I, II	AT, AF, AFib, PACs, SSNS, AVB I, II
Inheritance	Maternal grandfather – SCD, DCM (31 y.o) Maternal cardiac examination showed no pathology	No data	Brother had aborted cardiac arrest at 1 y.o. Maternal cardiac examination showed no pathology	No Similar mother's DNA pathology wasn't detected	Parents have no ECG pathology.
Current age	20 y.o.*	18 y.o.*	28 y.o.	22 y.o.	15 y.o.
Structural and functional cardiac abnormalities	LA dilatation (z-score** 3.04) and LV EF 54%	RA dilation (z-score 2.72),* RV dilation (z-score 2.98)	No	No	No
Antiarrhythmic therapy	Metoprolol tartrate, Lappaconitine hydrobromide, Sotalol, Propafenone	Lappaconitine hydrobromide	No	Metoprolol tartrate	Metoprolol tartrat, Propafenone
Other therapy	Perindopril	Perindopril Spironolactone	No	No	No
Clinical symptoms	Palpitations	Palpitations	Dizziness	Palpitations, reduced physical tolerance	Palpitations, reduced physical tolerance
Syncope	No	No	No	No	No
Neuromuscular phenotype	Not in childhood Mild myopathic changes in the upper limbs by ENMG after 18 y.o.	No	Not in childhood elbow contractures presented at 25 y.o	Myopathic changes since 1.5 y.o. Detected by ENMG	elbow and ankle contractures, muscle weakness in the upper limbs since 11 y.o.
Pacemaker implantation	16 y.o.	Patient's refusal	No	No	15 y.o.
RFA	AVNRT (eff) AF (eff)	Multifocus AT (no eff)	No	No	No
CK level	Normal	Normal	↑CKx2	↑CKx7	↑CKx6,5

*AT, atrial tachycardia; AF, atrial flutter; AFib, atrial fibrillation; AVB, atrioventricular block; AVNRT, atrioventricular nodal reentrant tachycardia; CK, creatine kinase; EF, ejection fraction; eff, efficiency; ENMG, electroneuromyography; LA, left atrium; LV, left ventricle; PACs, premature atrial captures; PVCs, premature ventricular contractions; RFA, radiofrequency ablation; SSNS, sick sinus node syndrome; RV, right ventricle; SAB, sinoatrial block; SB, sinus bradycardia*.

* *z-score values were used for assessment of dilatation by echocardiography*.

** *LV dysfunction was considered as a reduction of LV ejection fraction <55% (Simpson method)*.

### Patient 1

**Figure 1:**
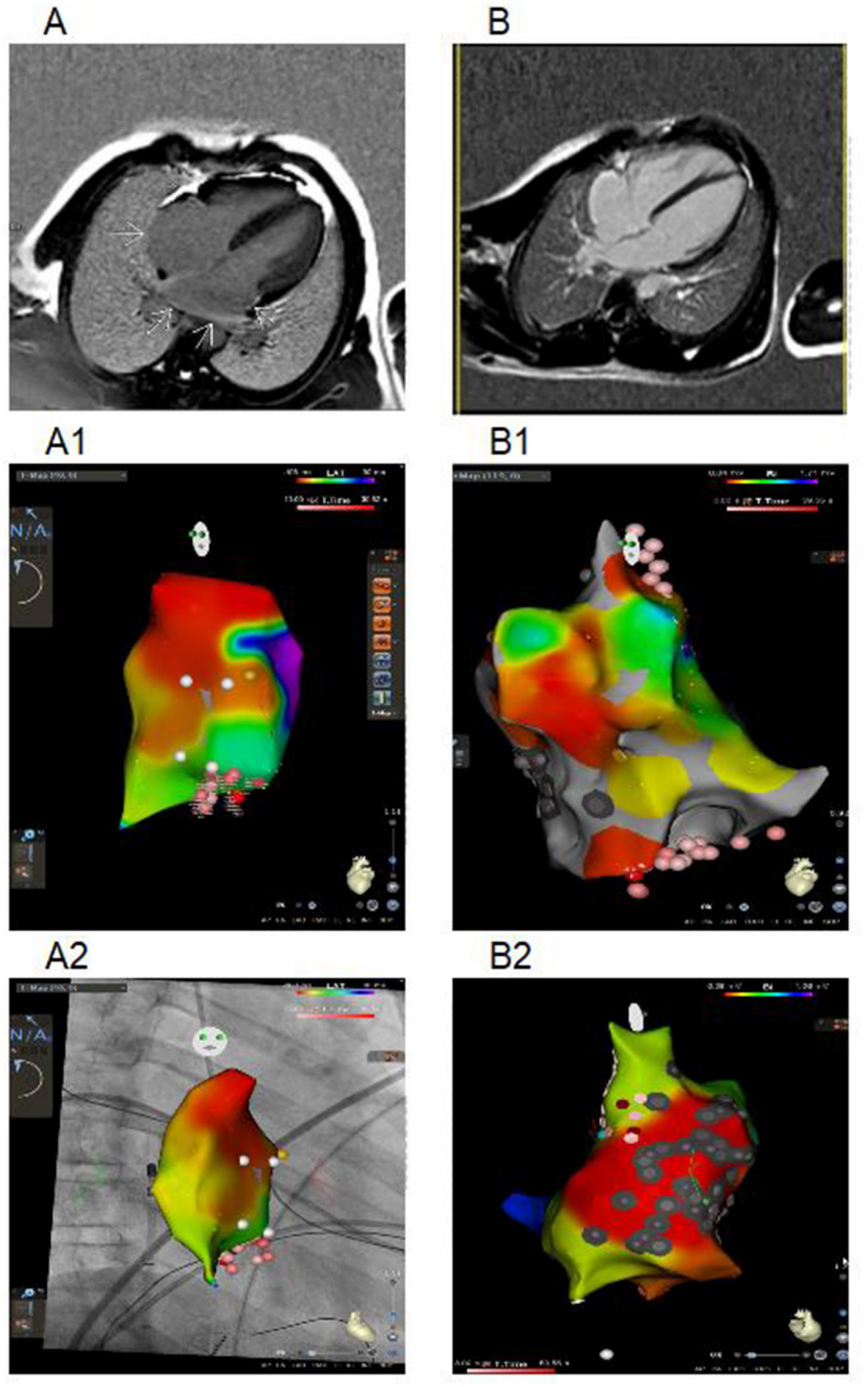
Cardiac maps using three-dimensional mapping system and CMR (imaging in the 4-chamber plane) of Patient 1 and 2. **(A)** CMR from a patient 1: delayed-enhancement CMR shows areas of myocardial scarring in both atria (arrows). **(A1)** Right atrial activation map (CARTO 3 system), LAO position. White dots – location points, yellow dots – part of conduction system, red and pink dots – RF ablation points (the degree of color intensity depends on the RF exposure time). **(A2)** Right atrial activation map (CARTO 3 system with CARTOUNIVU module), RAO position. White dots – location points; yellow dots – part of conduction system, red and pink dots – RF ablation points (the degree of color intensity depends on the RF exposure time). **(B)** CMR from a patient 2 with RA dilatation. **(B1)** Bipolar map of right atrium (CARTO 3 system), LAO position. **(B2)** Bipolar map of right atrium (CARTO 3 system), non-standard position with focus on the scar and ablation sites. Gray dots – scar points, white dots – location points, blue dots – double potential, red and pink dots – RF ablation points (the degree of color intensity depends on the RF exposure time). Bipolar color scale: red fill color has the lowest voltage.

**Figure 2:**
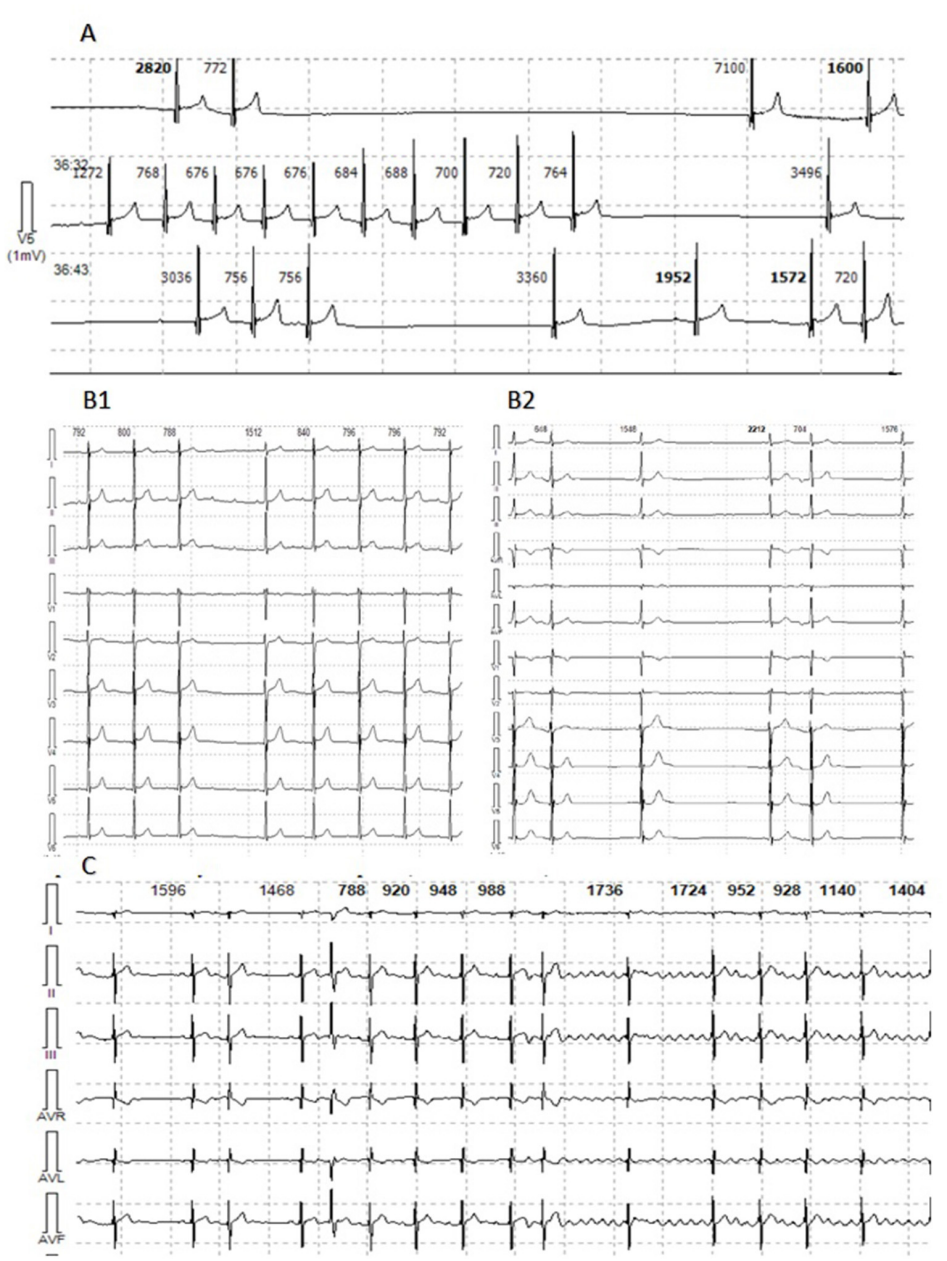
Holter monitor fragments of Patient 1, 2, and 5. **(A)** Patient 1. Sinus arrest with pauses from 3,036 to 7,104 ms. **(B1)** Patient 2. Episode of sustained AT with variable AV conduction. **(B2)** Patient 2. Multifocal PACs. **(C)** Patient 5. Episodes of multifocal PACs, AT and AFib with variable AV conduction.

The patient first presented with atrial rhythm and rare PACs on the ECG at the age of 14 years. He had no complaints, was a member of a football team and was undergoing ECG annually. No pathology was noticed on echocardiography. At 14.5 y.o. he complained on palpitations. An EPS revealed three types of arrhythmias: non-sustained mAT with heart rate (HR) 114 bpm, non-sustained AF and slow-slow AVRNT with HR 130 bpm. The radiofrequency ablation (RFA) of AVRNT was performed. Metoprolol tartrate was prescribed but canceled due to progressive AVB with pauses up to 3,261 ms. Six months later, AF with irregular AV conduction (2:1–4:1) was registered and followed by to RFA of the inferior vena cava-tricuspid isthmus. During the procedure, extensive low-amplitude areas corresponding to fibrosis fields were observed in the right atrium (Figure 1). CMR showed mild-dilated right atrium, atrial fibrosis and no acute inflammatory (Figure 1). The progression of the sinus and AV nodes dysfunction with bradycardia to 34–43 bpm and pauses to 7,104 ms (Figure 2), increasing atrial ectopy led to dual-chamber pacemaker (PM) implantation and Propafenone therapy. Increasing of pacing percentage, no intrinsic rhythm, non-sustained AFib during PM programming registered over time. Target genetic screening using 108-gene panel identified novel genetic variant in *EMD* gene (NM_000117.3):c.631delC, p.Arg211ValfsTer26 (chrX:153609422) classified as pathogenic according to ACMG criteria (PVS1, PM2, PP3). Parental genetic testing hasn't been performed. CK level was not elevated. Neurological examination showed no pathology, but mild myogenic changes in the upper limbs were detected by ENMG later, at the age of 18 years. Maternal grandfather died suddenly at the age of 31 with dilated cardiomyopathy according to the autopsy. Maternal cardiac examination revealed no pathology.

### Patient 2

Patient 2 was diagnosed with atrial rhythm with 93 bpm during ECG before being brought to the orphanage at the age of 11 years. His family history is unknown. He has been abusing nicotine and alcohol since the age of 15. On ECG, at the age of 14, atrial and junctional rhythm with PACs and non-sustained mAT were registered. Six months later, he was hospitalized in the emergency unit with palpitations. AT with HR to 200 bpm were registered and aborted by overdriving stimulation. Echocardiography and CMR confirmed right chambers dilatation without myocardial dysfunction. No signs of acute myocardial damage or fibrosis have been found (Figure 1). HM identified sinus bradycardia to 35 bpm, mainly atrial and junctional rhythm, mAT with variable AV conduction (2:1–7:1) and pauses to 4,404 ms (Figure 2). The RFA of mAT was performed (Figure 1) but wasn't effective. Endomyocardial biopsy showed no myocarditis signs. Target genetic screening using 172-gene panel identified *EMD* genetic variant (NM_000117.3):c.449+1G>A genetic variant, classified as pathogenic according to ACMG criteria (PVS1, PM2, PP3) previously reported in association with EDMD (38). He had not myopathy signs or contractures. ENMG was not performed due to the patient's unwillingness. CK level was normal. PM implantation was recommended, but the patient refused. Attempts to prescribe antiarrhythmic therapy (AAT) were unsuccessful.

### Patient 3

Patient 3 first consulted a cardiologist at the age of 16 because of dizziness and fatigues. No structural abnormalities were detected by echocardiography. Rare symptomatic episodes of resting bradycardia to 30–34 bpm and pauses due to AVB II up to 2,500 ms were observed. Neurological examination didn't reveal any pathology. Later, at the age of 25, mild elbow contractures were noted. Two times higher CK level was documented at the age of 28. Genetic screening identified a variant in *EMD* (NM_000117.3):c.173C>T (p.Ser58Phe), classified as a variant of unknown significance according to ACMG criteria. Brother survived the aborted cardiac arrest at the age of 1 year, but his genotyping is currently infeasible. Maternal cardiac examination showed no pathology.

### Patient 4

The patient was first referred to a neurologist at 1.5 years of age due to toe-walking. He had two times elevated CK level and myopathic changes detected by ENMG. Multifocal PACs were registered at the age of 16 years when the fatigue and palpitations appeared. A HM showed paroxysms of non-sustained AFib, multiple PACs, and transient AVB II and SA block. No pathology was detected on echocardiography and CMR. The CK level has been increased seven-fold. He had ankles contractures, muscle weakness in the shoulders and lower legs. Metoprolol tartrate was initiated with good response and without conduction worsening. Three months later, atrial arrhythmias were increased and rare premature ventricular contractions (PVCs) were registered. Metoprolol tartrate dose was doubled with a positive effect. The genetic investigation identified *LMNA* (NM_170707.4):c.746G>A (p.Arg249Gln) variant previously described in EDMD patients (rs59332535) and classified as pathogenic according to ACMG criteria. Mother doesn't have this variant, father died at 49 y.o. during a planned surgery due to cardiac arrest, no DNA was available for the genotyping. This patient has not indications for PM or cardioverter-defibrillator implantation (ICD).

### Patient 5

**Figure 3:**
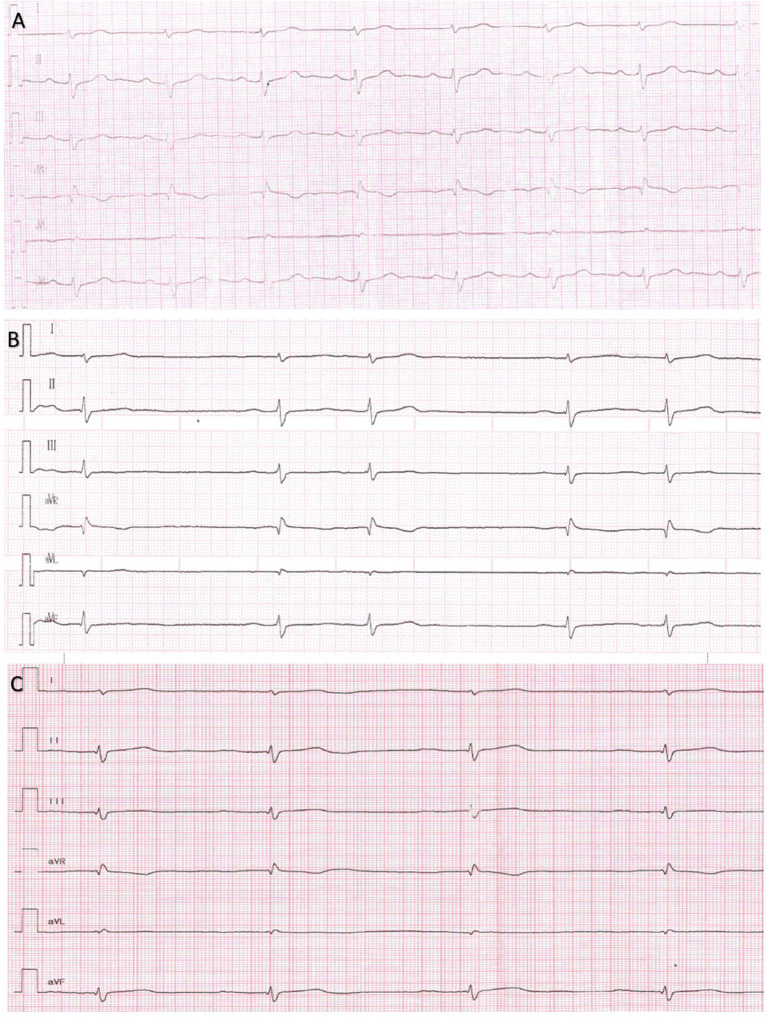
Series of Electrocardiograms from a patient 5 showing reduced amplitude of P wave and AV conduction in dynamics. **(A)** 13 y.o. P wave amplitude 2.4 mm, P wave duration 70 ms, PQ 160 ms. **(B)** 14 y.o. P wave amplitude 1.5 mm, P wave duration 100 ms, PQ 200 ms. **(C)** 15 y.o. P wave amplitude 1 mm, P wave duration 110 ms, PQ 250 ms.

The patient was first examined by a cardiologist at nine y.o. due to palpitations, but ECG revealed no pathology, HM and echocardiography were not performed. At the age of 11 episodes of mAT, PACs, and transient AVB I were detected by HM. Concurrently, elbow contractures and muscle weakness were noted. ENMG identified a moderate myopathic pattern in the lower limbs. Increase in CK by 6.5 times was detected. Within the next several years, arrhythmias progressed with the sustained episodes of mAT, AFib, deterioration of AV conduction and decrease of HR. Specific changes in the P-wave were detected (Figure 3). No structural abnormalities were detected by echocardiography. Metoprolol tartrate was up-titrated with a good but temporary effect. The genetic study identified an earlier reported variant in the *LMNA* gene (NM_170707.3):c.305T>C, (p.Leu102Pro). Family history was unremarkable. PM was implanted along with AAT (Propafenone) at the age of 15 years.

## Discussion

Supraventricular tachycardia and conduction disorders with sinus and AV node dysfunction are the most common conditions in pediatric cardiology practice. The thorough diagnostic workup and precise nosology definition are important for the optimal therapeutic strategy. The presented cases as well as several earlier reports (Table 2) provide the evidence that EDMD has to be considered in children presenting with conduction disorders or atrial arrhythmias even without overt signs of muscular dystrophy and joint contractions. The natural cause of EDMD leads to the requirement of PM in 56% of the patients, AF/flutter developed in 61%, atria standstill in 45%, and embolic stroke in 36% of the patients (18). Therefore, identification of EDMD-underlying genetic background is important for following genetic cascade screening first-degree relatives of patients, that is recommended and includes ECG, HM, echocardiography, and CMR (43).

**Table 2:** Literature review on heart rhythm disturbances in EDMD in childhood.

**References**	**Year**	**N of patients**	**Gene/Sybtype**	**Age of onset**	**Age of cardiac onset in childhood**	**Muscle weakness**	**Contractures**	Cardiac involvement *in childhood
Bonne et al. (39)	2000	53 (6 families +17)	LMNA/ EDMD 2	0–40	14.7 ± 2.12 (from 11 to 17)	41 (77.3%) *40 (75.4%)	41 (77.3%)	All aged groups 41 (77.3%), incl. Isolated: 12 (29%) *7 (13.2%), incl. 1 isolated Arrhythmias - 5 Arr. + VD - 1 1 – n/a
Vohanka et al. (14)	2001	1	EMD/ EDMD 1	16	16	+/–	–	Atr. standstill AVB PM at 26 y.o. Afib at 27 y.o.
Sanna et al. (11)	2003	10	LMNA/ EDMD2 EDMD3	3.4 ± 1.9	14.1 ± 1.8	7 (70%)	10 (100%)	8 (80%) *6 (60%) AF – 2; AFib – 1; AT – 3; PACs – 2 NsVT – 3 AVB – 2; BBB – 3 SSD – 3; Atr. standstill – 1 Junct. rhythm - 1 Mild LV dil - 1
Hong et al. (40)	2005	3	EMD, LMNA/ EDMD 1 EDMD 2	1, 3, 15	14 (EDMD 1)	3 (100%)	2 (66.6%)	3 (100%)/*1 Atr. standstill, AVB, SSD, Junct.rhythm, PM, RV&RA dil
Sakata et al. (34)	2005	33 (16 carriers) *3	EMD/ EDMD 1	n/a *10, 13, 14	10, 14	*1 (6.25%)	3 (18.8%) *2	10 (62.5%) *3 (18.8%) SSD, AVB, PM - 1 RA dil - 2
Karst et al. (33)	2008	10 (1 family) *4	EMD/ EDMD 1	4 –teens 4 – from 28 to 31 y.o.	Teens	–	–	8 (80%) *4 (40%) Arrhythmias – 4 Syncope - 1
Nigro et al. (41)	2010	1	EMD/ EDMD 1	5	10	+	–	SSD, Junct.rhythm, AVB, PACs, VT
Homma et al. (42)	2018	1	LMNA/ EDMD 2	3	5	+	+	AF
Fan et al. (10)	2020	84 32 – EDMD 11 - LGMD1B	LMNA/ EDMD2	EDMD: 2.2 ± 1.7 LGMD1B: 2.6 ± 3.0	indicated in column “Cardiac involvement”	17 (53.1%)	24 (75%) av. age 9.6 2 (18.2%) av. age 20.5	14 (43.8%) + 1 (9.1%) ST – 4 (av. age 6.9) + 1 (16) PACs – 1 (11) AVB – 1 (14) HF, PH, SSS – 1 (17)
Our study	2021	5	EMD, LMNA/ EDMD 1 EDMD 2	10.3 ± 4.4	13.2 ± 3.11	2 (40%)	3 (60%)	5 (100%) SVT – 3; AF – 1; AFib – 3 PACs – 4; PVCs – 1 SSD – 5; Junct.rhythm – 1 AVB – 4 PM – 2 RA dil – 1

*Arr, arrhythmias; AT, atrial tachycardia; AFib, atrial fibrillation; AF, atrial flutter; AVB, atrioventricular block; BBB, bundle brunch block; HF, heart failure; mild LV dil, left ventricular dilatation; NsVT, non-sustained ventricular tachycardia; PACs, premature atrial captures; PH, pulmonary hypertension; SSD, sick sinus disfunction; ST, sinus tachycardia; VD, ventricular disfunction. Bold indicates the number of children in the study with cardiac involvement*.

* *Onset in childhood*.

The precise molecular mechanisms underlying predominantly atrial and conduction pathology in EDMD are largely unknown. Most of the genes and genetic loci identified by GWAS linked to AFib represent transcriptional factors and signaling molecules responsible for cardiac development and cardiomyocyte differentiation (44). In this context, the ability of nuclear envelope proteins to modify chromatin organization and transcriptional activity could explain the effect of *EMD* deficiency on myocardial cell fate (45, 46). A deficiency or defects of this proteins could lead to nuclear instability in tissues undergoing mechanical stress, including cardiac and skeletal muscle. Of note, another nuclear envelope protein – nesprin 2 encoded by the *SYNE2* gene and reported as a rare cause of EDMD was also identified among GWAS loci in association with AF (47, 48). In addition, Shimojima et al. reported that mutant forms of emerin caused abnormalities in nuclear Ca^++^ transients, which may further modulate nuclear transcriptional pathways in response to mechanical stress (49). As a result, alterations in cardiomyocyte differentiation gradually led to replacing the normal myocardial cells with fibrotic tissue leading to sinus node dysfunction, ectopic loci, and conduction defects. The question remains why these processes mainly start in the atria, often involve the atrioventricular node and only eventually affect the ventricles. This question is further difficult to address because of the unavailability of atrial tissue for morphological examination and limitation of CMR for atrial imaging because of difficulties in achieving adequate image resolution in thin-walled atria (50).

Cardiac structural abnormalities, ventricular arrhythmias and dysfunction are more common in AD-EDMD and are not typical of X-linked EDMD. Of note, in the presented case series, the only patient who demonstrated rare PVCs was the Patient 4 with *LMNA*-associated EDMD. Together with other reports on *LMNA*-associated SCD in patients with cardiac and muscle pathologies, this further draws attention for the more thorough follow-up of this group in terms of different arrhythmias (51).

It is commonly accepted that cardiac symptoms of EDMD follows the neuromuscular phenotypes and becomes evident in the second-third decade of life (7, 11, 23). Cases of isolated cardiac manifestations of EDMD remain rarely reported, especially in pediatric patients (7, 33, 40). However, such cases, together with our study, emphasize the importance of genetic testing and target search for EDMD in cases of progressive refractory arrhythmias with or without specific neurological and laboratory findings. Three patients with EDMD1 we observed did not have a typical clinical course with early contractures or muscle weakness, but two patients had severe cardiac abnormalities requiring medication and intervention treatment. Patients 4 and 5 with *LMNA*-genetic variants demonstrated the clinical variability of EDMD ranging from mild disease course and later cardiac debut to early manifestation and fast-progression that led to PM and AAT. Thus, the results of genetic testing helped us draw up a plan of management and further follow-up, including annual cardiological and neurological examinations.

The main therapeutic problem we faced was coexistence of symptomatic high-frequency atrial arrhythmias and conduction disorders, which limited the prescribing of AAT. The use of RFA in EDMD is a debatable issue (52). The literature search resulted in four cases of RFA in EDMD described in details: three with poor outcomes [1 – RFA of AF with recurrence of arrhythmia, leading to systolic dysfunction and heart transplantation (53); 2 – recurrent SVT with PM after AV nodal ablation, accession of ventricular tachycardia and death from irrecoverable asystole (54); 3 – repeated unsuccessful RFA of AFib leading to embolic stroke (42)] and one successful: RFA of AF by employing a three-dimensional mapping (52). In our study, RFA was performed in two patients: successful treatment of AVNRT and AF in Patient 1 and unsuccessful RFA of mAT in Patient 2.

Since sudden death in X-linked EDMD is primarily caused by a complete heart block, it can be reliably averted by PM implantation (3). The ICD, cardiac resynchronization therapy, mechanical circulatory support, and heart transplantation remain a rare but potentially applicable strategies in EDMD patients (21, 43, 55). In the long-term longitudinal study performed by Boriani et al., heart failure requiring transplantation occurred in 6% and asymptomatic LV dysfunction in 17% of patients (18). ICD is more often required for *LMNA* mutation carriers and should be considered with sustained or non-sustained ventricular tachyarrhythmias, especially in those with LVEF <45% or in patients with indications for PM implantations (41, 56). In line with that, three of five described patients had I class indications for PM, and none had indications for ICD implantation.

## Conclusion

In conclusion, while being rare cases, heart rhythm disorders can represent the first and for a long time, the only clinical symptom of EDMD even in the pediatric group of patients. Therefore, thorough laboratory and neurological screening along with genetic studies are of importance in each pediatric patient presenting with complex arrhythmias of primary supraventricular origin to exclude EDMD or other neuromuscular disorders. Consideration of EDMD in diagnostic workup can facilitate the optimal strategy and personalized follow-up of this group of patients.

## Data Availability Statement

The data presented in the study are deposited in the Gene bank repository, accession numbers SCV001548550–SCV001548554 and publicly available.

## Ethics Statement

The studies involving human participants were reviewed and approved by Almazov National Medical Research Centre Institute Ethical Review Boards. Written informed consent to participate in this study was provided by the participants' legal guardian/next of kin.

## Author Contributions

EY, SF, and TL contributed to the conception and design of the study, analysis, and interpretation of the data and drafting of the manuscript. TK contributed to the study concept and research design and wrote the manuscript. AKos, TP, and EV made contributions to the conception and design of the study and revision of the manuscript critically. VL, TV, DL, and LM took part in the analysis and interpretation of the data and have been involved in revising the manuscript critically. AR, PS, YF, AKoz, SZ, NS, and AZ conducted the experiments and performed the analysis and interpretation of the data. All authors have read and agreed to the published version of the manuscript.

## Conflict of Interest

The authors declare that the research was conducted in the absence of any commercial or financial relationships that could be construed as a potential conflict of interest.
