# Supplementary figures and images for: Case Reports: Emery-Dreifuss Muscular Dystrophy Presenting as a Heart Rhythm Disorders in Children
Source: Front Cardiovasc Med. 2021 May 7;8:668231. doi: 10.3389/fcvm.2021.668231 (PMC8137911; doi:10.3389/fcvm.2021.668231)

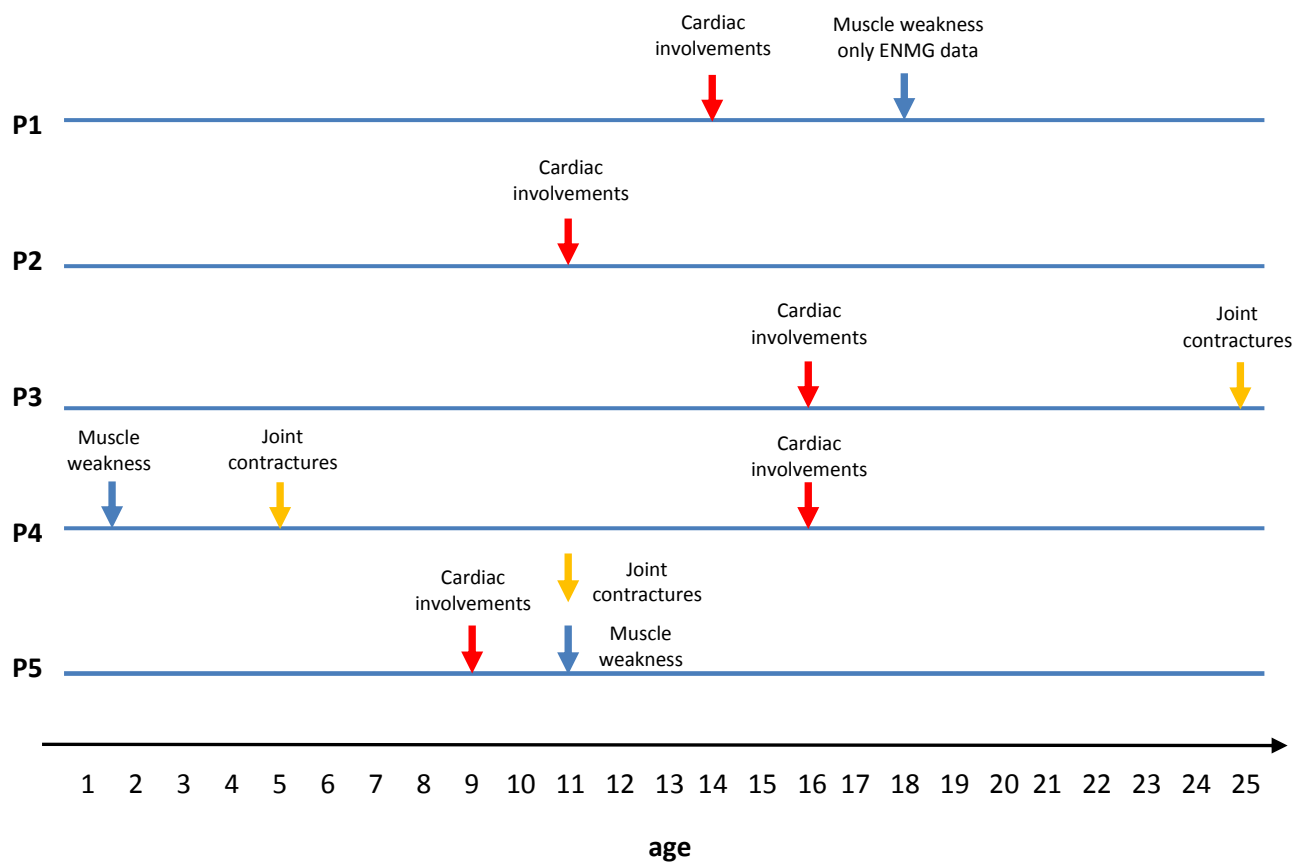

Supplement: Supplementary file 2 [file Data_Sheet_1.PDF]
